# Pathogenicity and Whole Genome Sequence Analysis of a Pseudorabies Virus Strain FJ-2012 Isolated from Fujian, Southern China

**DOI:** 10.1155/2017/9073172

**Published:** 2017-12-31

**Authors:** Xue-min Wu, Qiu-yong Chen, Ru-jing Chen, Yong-liang Che, Long-bai Wang, Chen-yan Wang, Shan Yan, Yu-tao Liu, Jin-sheng Xiu, Lun-jiang Zhou

**Affiliations:** ^1^Institute of Animal Husbandry and Veterinary Medicine, Fujian Academy of Agriculture Sciences/Fujian Animal Disease Control Technology Development Center, Fuzhou 350013, China; ^2^College of Animal Sciences, Fujian Agricultural and Forestry University, Fuzhou, Fujian 350002, China

## Abstract

The outbreaks of pseudorabies have been frequently reported in Bartha-K61-vaccinated farms in China since 2011. To study the pathogenicity and evolution of the circulating pseudorabies viruses in Fujian Province, mainland China, we isolated and sequenced the whole genome of a wild-type pseudorabies virus strain named “FJ-2012.” We then conducted a few downstream bioinformatics analyses including phylogenetic analysis and pathogenic analysis and used the virus to infect 6 pseudorabies virus-free piglets. FJ-2012-infected piglets developed symptoms like high body temperature and central nervous system disorders and had high mortality rate. In addition, we identified typical micropathological changes such as multiple gross lesions in infected piglets through pathological analysis and conclude that the FJ-2012 genome is significantly different from known pseudorabies viruses, in which insertions, deletions, and substitutions are observed in multiple immune and virulence genes. In summary, this study shed lights on the molecular basis of the prevalence and pathology of the pseudorabies virus strain FJ-2012. The genome of FJ-2012 could be used as a reference to study the evolution of pseudorabies viruses, which is critical to the vaccine development of new emerging pseudorabies viruses.

## 1. Introduction

Pseudorabies virus (PRV), also called Aujeszky's disease virus or *Suid herpesvirus* 1, is the causative agent of pseudorabies (PR), which infects a wide variety of animals from mollusks to mammals and damages world economy. PRV is a member of the subfamily Alphaherpesvirinae in the family Herpesviridae belonging to the genus *Varicellovirus* [1]. Though the virus was first described in cattle by Aujeszky in 1902, pigs are the natural reservoir for PRV [2, 3]. The clinical symptoms of PR in pigs are characterized by central nervous system (CNS) disorders in piglets, abortion in pregnant swine, and respiring signs in older pigs [4].

The PRV genome encompasses a unique long segment (UL) and a unique short region (US) flanked by the internal and terminal repeat sequences (IRS and TRS, resp.), encoding more than 70 proteins. The virulence of PRVs and the immunology mutual protection between them are determined by multiple genes, and thus the genome-wide analysis is necessary to define all the characteristics of the viruses [5, 6].

Pseudorabies had been well controlled in China due to the wide usage of gE-deleted vaccines and the serum distinguish test [7]. However, in late 2011, outbreaks of PR were reported in Bartha-K61-vaccinated farms, and the disease rapidly spread to 11 provinces from northern to eastern China including Heilongjiang, Jilin, Liaoning, Tianjin, Jiangsu, Zhejiang, and Fujian. A few studies showed that the current PR outbreaks on farms were caused by PRV variants, and the PR vaccine could not provide effective protection against the prevalence of PRV strains in China [8–10]. However, the complete genomes of the variants and their molecular characteristics are unclear, which is an obstacle in producing effective vaccines. As a result, the outbreaks have caused a great economic loss to swine-feeding industry in China.

In this study, we thoroughly assessed a pseudorabies virus named “PRV FJ-2012,” which was isolated from a Bartha-K61-vaccinated pig farm in Fujian Province during a PR outbreak. The outbreak has the following characteristics: (1) the mortality of infections can be as high as 100% and (2) plenty of pregnant sows aborted. We have characterized the pathogenicity in pigs and analyzed the complete genome of the PRV FJ-2012 in order to characterize its molecular properties and virulence.

## 2. Materials and Methods

### 2.1. Virus, Cells, and Genomic Viral DNA Preparation

PRV FJ-2012 was isolated from pig brain samples collected from a Bartha-K61-vaccinated farm with a PR outbreak in Fujian Province, southern China, in 2012. The virus was determined to be a pseudorabies viral strain by the PCR analysis and the sequence analysis of its partial gE gene. Virus was propagated on PK-15 cells, cultured in Dulbecco's modified Eagle's medium (DMEM, Hyclone, USA) containing 1% fetal bovine serum (FBS, Gibco, USA), 100 IU/mL penicillin, and 100 μg/mL streptomycin at 37°C and 5% CO_2_. Cells were harvested when the cytopathic effect (CPE) of PK-15 cells that were inoculated by PRV FJ-2012 strain reached 80%. After freeze-thaw for three times, the cell debris was removed by centrifugation at 5000 ×g for 30 min at 4°C. Then, the supernatant involving PRV was centrifuged by a Beckman ultracentrifuge (LE-80K) at 30,000 ×g for 2 h at 4°C; the supernatant was discarded, and the pellets were then resuspended in 2 mL PBS (0.01 mol/L, PH7.2). Discontinuous mass fraction sucrose gradients (30%, 35%, 40%, and 45%) that were formulated with PBS were further purified at 26,000 ×g for 2 h at 4°C. And then, the virus band (between 35% and 40%) was drawn to a centrifuge tube, and the sucrose was removed by centrifugation at 30,000 ×g for 1 h at 4°C. The purified virus particle was obtained and used to prepare genomic viral DNA using QIAamp DNA Mini Kit (QIAGEN, Germany) according to the manufacturer's instructions.

### 2.2. Experimental PRV Inoculation of Pigs

Ten healthy, 28-day-old Duroc × Landrace × Yorkshire (DLY) hybrid pigs were collected from PRV-free swine farm and confirmed to be serologically negative for PRV antibodies with a gB ELISA kit (IDEXX, USA). The pigs were randomly allocated to two groups, namely, the challenge and control group, and each group was housed in separate pens. Four pigs in Group 1 (the challenge group) were each inoculated intranasally (i.n.) with 1 mL 10^6^ TCID50 FJ-2012 strain, and the other two pigs in Group 1 were i.n. with DMEM with the same dose as the cohabit infective test. The remaining four pigs were served as uninfected control. Clinical signs and rectal temperatures of pigs were recorded daily throughout the study. At 14 dpi, all surviving pigs were euthanized.

### 2.3. Tissue Sampling and Histological Analysis

After macroscopic examination, the tissue samples required for histological examination were obtained from the brains, kidneys, lungs, tonsils, livers, and lymph nodes (superficial inguinal). These samples were fixed in 10% formalin, processed routinely, and embedded in paraffin. Each paraffin sample was sectioned to 4-5 μm and stained with HE. The sections were viewed with a Motic BA210 microscope.

### 2.4. Genomic Sequencing, Assembly, Annotation, and Analysis

The genome of PRV FJ-2012 strain was sequenced with a Pacific Biosciences RS II sequencer (Pacific Biosciences, Menlo Park, CA, USA), using a single-molecule long-read sequencing technology with a 10K SMRTbell template library. We are fully aware that the long reads from the Pacific Biosciences RS II sequencer have some disadvantages like high error rate compared to short reads. However, we still prefer long reads since they are better at identifying gene isoforms and assembly, which are critical to this study. We then removed host reads by comparing the reads against the pigs (*Sus scrofa*) using BLAST. The remaining reads were de novo assembled; the obtained contigs were assembled with Celera software (https://sourceforge.net/projects/wgs-assembler/files/wgs-assembler/wgs-8.3/), and the scaffolds were constructed by comparing the contigs with reference PRV genomes (GenBank Accession # NC_006151) using the NCBI BLAST program. Finally, a Perl program was used to check gaps between the scaffolds, and the alignments were extremely high coverage with no gap.

The open reading frames (ORFs) of FJ-2012 genome were searched by ORF Finder (http://www.ncbi.nlm.nih.gov/gorf/), and genes were predicted and analyzed by GeneMarkS [11]. The ORF annotations of the FJ-2012 strain were created by BLAST homology-based transfer as previously described in [5, 12, 13]. The alignments were performed using the mVISTA genomic analysis tool with a LAGAN global alignment [14]. The phylogenetic trees were constructed using the neighbor-joining algorithm with 1000 bootstrap repetitions using the Kimura 2-parameter substitution model in MEGA 5.0 [15–17]. The distribution of polymorphic sites in eight PRV genomes was inferred using the software Base-By-Base [18].

## 3. Results

### 3.1. Pathogenicity of the PRV Strain FJ-2012 in Pigs

After intranasal infection of the 28-day-old PRV-free piglets, all pigs in the challenge group developed high fever beginning at 2 dpi with temperatures 41.9°C–42.5°C. The clinical signs were consistent with typical pseudorabies syndrome, from listlessness, anorexia, to high fever and then displayed respiratory symptoms such as cough, sneeze, and central nervous system (CNS) symptoms. Finally, all pigs in the challenge group exhibited opisthotonus and were dead in 8–14 dpi. The pigs in the cohabitation infection group also showed high fever with temperatures 41.9°C–42.5°C, but the time of onset was 4 days later and the clinical signs were milder than those in the challenge group. The pigs were also dead in the end (Table 1). In contrast, pigs in the control group remained healthy without any abnormal symptom.

### 3.2. Gross Lesions

The pigs in the FJ-2012-infected group showed multiple gross lesions. Macroscopic encephalic hemorrhages or encephalemia was observed in all pigs (4/4). Three of those pigs (3/4) showed pinpoint hemorrhages in the kidney. Small white foci were seen in the liver of four pigs (4/4). Diffuse reddened foci and edema of the lungs were observed in all pigs (4/4). The dark-red hemorrhage and congestion were noted in the lymph node of four pigs (4/4). Tonsil anabrosis was observed in three pigs (3/4). Two pigs in the cohabitation infection group showed milder lesions (such as slight hemorrhages in the brain and lung). No pig in the control group displayed gross lesions.

### 3.3. Histopathological Analysis

**Figure 1:**
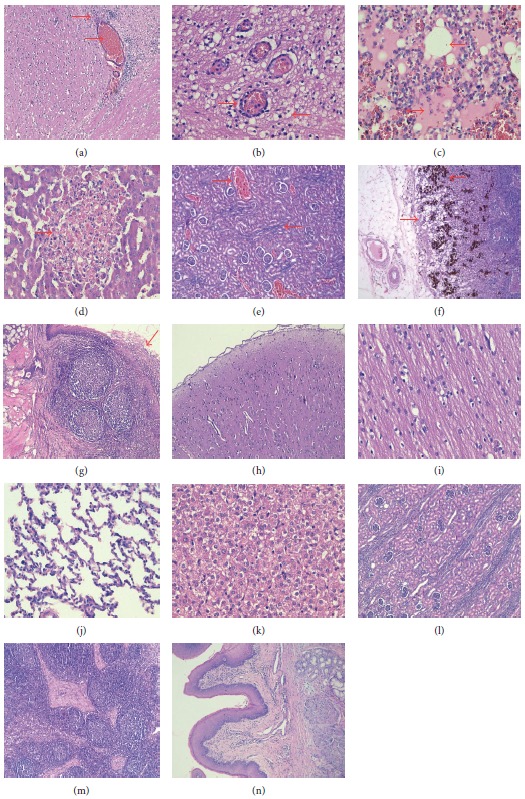
Histopathological findings of PRV-infected or control pigs. The pictures show representative tissue samples from the PRV-infected (a–g) and control (h–n) groups stained with H&E, 100x (a and h) and 400x (b–g, i–n). Brains (a, b, h, and i), lungs (c and j), livers (d and k), kidneys (e and l), lymph nodes (f and m), and tonsils (g and n).

To further study the pathology of these organs, samples from brains, kidneys, livers, lungs, lymph nodes, and tonsils of pigs were stained with hematoxylin and eosin (H&E). On one hand, the histopathological examination showed multiple lesions in several organs of PRV-infected pigs. For example, nonsuppurative ganglioneuritis, characterized by gliosis, hemorrhage (Figure 1), pronounced perivascular inflammatory infiltrates, and nerve cell necrosis were observed in the brain (Figure 1). The lungs showed severe hemorrhage, congestion, and edema, and bronchiolar cavities were filled with cellular serous exudates that were red-stained (Figure 1). Multiple small focal necrosis were discovered in the liver (Figure 1) and the kidney with lymphocytic infiltration and congestion (Figure 1). The striking changes in the lymph nodes include brownish-stained hemosiderosis and lymph follicle swelling (Figure 1). The stratified squamous epithelium of tonsil appeared modified, necrosed and exfoliative (Figure 1). On the other hand, the histopathological results of these organs from the control group had no significantly pathological changes (Figures 1–1).

### 3.4. Complete Genomic Characterization of the PRV FJ-2012 Strain

**Figure 2:**
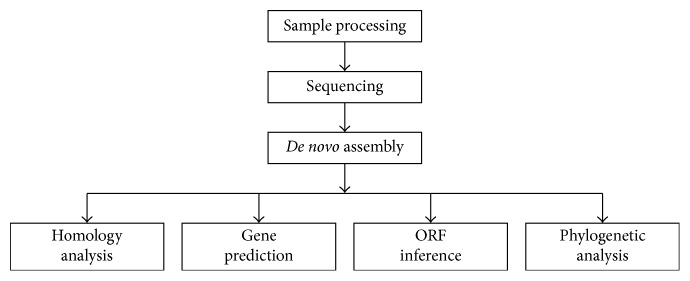
Data analysis flow for FJ-2012 sequence.

We performed the whole genome sequencing of FJ-2012 and performed a few downstream analyses on the sequencing data (Figure 2). The total length of its genome is 144,873 bp with a high G + C content of 73.5%. The overall genomic composition is the same as that of a few previously studied strains (Bartha, Kaplan, and Becker), consisting of a unique long (UL) region (position at 1–102,119), a unique short (US) region (118,893–128,096), internal repeat sequences (IRs, 102,120–118,892), and terminal repeat sequences (TRs, 128,097–144,873), which are located at the flank of the US region (Figure 1). 70 open reading frames (ORFs) were identified, which relate to 70 genes that encode 68 different proteins (Figure 1) since there are the two putative genes (US1 and IE180). It is of note that US3 and US3.5 were treated as different genes due to their distinct functions [19].

The unique long regions containing 59 genes, most of which are involved in DNA replicative mechanisms and virus particle assembly and mature, are transcribed in both directions. Unique short regions containing 7 genes transcribed backward are likely affecting pathogenesis and host range functions. IRS and TRS containing US1 and IE180 genes, which function as an accessory regulatory protein and interact with the IE protein to enhance gene transactivation, are transcribed in reverse directions [20].

### 3.5. Comparison and Phylogenetic Analysis among PRV Strains

We compared the genomes of FJ-2012 with those of other 7 PRV strains including Kaplan, Becker, Bartha, JS-2012, HeN1, TJ, and ZJ01. The four strains JS-2012, HeN1, TJ, and ZJ01 were isolated from China (Table 2). As can be seen, FJ-2012 exhibited 94.2% to 98.32% nucleotide identity with other strains from China and 89.8% to 91.9% nucleotide identity with strains outside China. Specifically, FJ-2012 is highly homologous to TJ strains (98.32%) and shared only 89.8% identity with the strain Bartha, which is deemed as an excellent vaccine strain to control pseudorabies.

**Figure 3:**
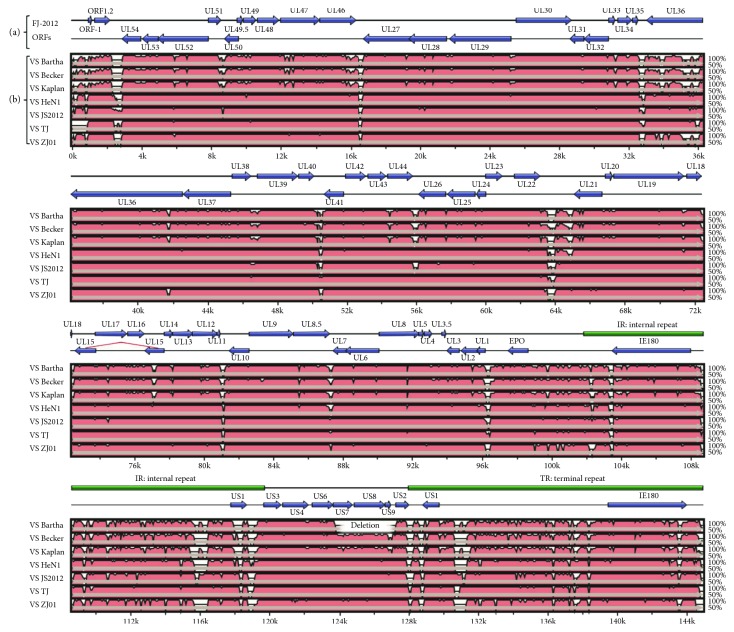
Genome organization of the PRV FJ-2012 and comparison of sequence conservation with other strains. (a) ORFs (horizontal bars in azure color) together with internal and terminal repeats (horizontal bars in green color) are depicted along with the genome. (b) Plots showing sequence conservation among PRV Bartha, Becker, Kaplan, HeN1, JS-2012, TJ, and ZJ01. Gene conservation was determined from a multiple sequence alignment, and the conservation score between any 2 genomes is plotted in a sliding 100 bp window.

In addition, most genetic variations among the PRV strains are located in noncoding regions, internal repeat sequence regions, and terminal repeat sequence regions (Figure 3 and see Table S1 available online at https://doi.org/10.1155/2017/9073172). There are also variations in a few coding regions such as US1, UL36, gN, gB, and gC. There are overall 11,893, 12,621 and 14,956 genomic changes, including 3759, 3724, and 3789 single-nucleotide substitutions between FJ-2012 and three non-China-origin viruses Kaplan, Becker, and Bartha, respectively (Table 2). The genomic changes between FJ-2012 and non-China-origin viruses are higher than those between FJ-2012 and China-origin viruses. Specifically, there are 4649, 4628, 2445, and 8557 genomic changes, including 716, 560, 206, and 1025 single-nucleotide substitutions between FJ-2012 and four China-origin viruses JS-2012, HeN1, TJ, and ZJ01, respectively.

**Figure 4:**
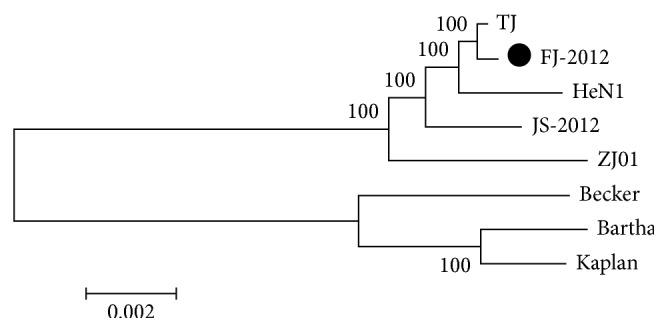
Phylogenetic comparison of PRVs. The sequences were aligned by Clustal, and the phylogenetic tree was constructed by MEGA 5.0 software using the neighbor-joining method with 1000 bootstraps. The nucleotide substitution model was chosen to be the Kimura 2-parameter substitution model.

We then performed the phylogenetic analysis of the 8 viruses based on their full-length genome sequences, which indicates that the PRV strains can be separated into 2 major groups corresponding to their geographic locations, namely, group genotype I consisting of strains in China and group genotype II consisting of European and American strains (Figure 4). FJ-2012 is phylogenetically most close to TJ strain.

### 3.6. Distribution of Polymorphic Sites and Protein Coding Variations of PRVs

**Figure 5:**
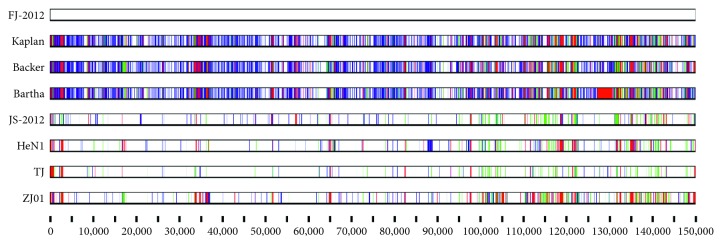
Distribution of polymorphic sites in eight PRV genomes. The genomes were aligned with CLC Genomics Workbench 8 and using Base-by-Base software. The sequence of the PRV strain FJ-2012 was used as the reference. Nucleotides in other 7 pseudorabies virus strains differing from the sequence of the PRV field isolate strain FJ-2012 are displayed: blue, nucleotide substitutions; green, insertions; and red, deletions.

We used Base-by-Base software to infer the distribution of polymorphic sites among the 8 PRVs. The changes between the genomic sequences of PRV FJ-2012 and those of non-China-origin PRV strains are predominantly substitutions, with a few insertions and deletions. However, the changes between FJ-2012 and China-origin strains JS-2012, HeN1, TJ, and ZJ01 are mainly insertions and substitutions (Figure 5). The sequence comparison revealed that PRV FJ-2012 genomes show great variations with other PRVs.

To further study the FJ-2012 genome, we compared its protein-coding regions with those of other viruses using local BLAST in BioEdit. The viruses in the group genotype I share 100% sequence identity with FJ-2012 for 16 ORFs and 94.7–99.9% identity for 53 ORFs. It is of note that HeN1 only shares 22.1% sequence identity with other strains on the protein IE180 due to a frame shift by upstream substitution. As for European and American strains (genotype II), FJ-2-12 shares 90.8–99.9% sequence identity in 69 ORFs (Table 2). The sequence alignment showed that FJ-2012 displayed extensive variations with previously isolated PRV strains including Kaplan, Becker, and the vaccine strain Bartha in most viral proteins, such as glycoproteins, for example, gN (UL49.5), gC (UL44), gL (UL1), gE (US8), and gB (UL27), tegument proteins, for example, UL36, UL46, UL16, and UL13, and nonstructural proteins, for example, IE180 and UL52 (Figure 5).

## 4. Discussion

Previous studies suggest that pseudorabies virus (PRV) can cause serious disease to piglets. The mortality rate is up to 100% for infected two-week old piglets with neurologic symptoms, while that for weaned piglets, the mortality rate is about 50% [19]. In this study, we tested the pathogenicity of FJ-2012 using six 28-day-old PRV-free piglets, four of which were in the challenge group and the other two were in the cohabit group. The piglets in the challenge group received intranasal challenge with 1 mL 10^6.0^ 50% tissue culture infecting dose, and those in the cobabit group were inoculated intranasally with DMEM with the same dose. Unfortunately, all 4 piglets in the challenge group died on day 8, 10, 12, and 14 post infection (DPI), respectively, and one piglet in the cohabit group died on 12 DPI (Table 1). The clinical symptoms of the piglets in the challenge group were consistent with those of typical pseudorabies syndromes including listlessness, anorexia, and high fever and respiratory symptoms such as cough, sneeze, and central nervous system symptoms. In postmortem and histopathological examinations of dead piglets, multiple lesion sites were observed in several organs (Figure 1). The findings suggest that FJ-2012 strain can cause severe pathological changes, which are more serious than previously observed [19].

In general, the virulence of virus and the immune failure are potentially associated with gene mutations. Previous surveys have showed that gene variants of isolated viruses contributed to the epidemics of PRV [8–10, 21]. Therefore, it is important and necessary to further analyze the genetic variations of the current PR for disease control and surveillance. Here, we have sequenced the whole genome of the PRV strain FJ-2012 by a Pacific Biosciences RS II sequencer, and the resulting sequences were assembled, predicted, and annotated of genes. The resulting PRV FJ-2012 genome sequence is 144,873 bp in length with a high G + C content of 73.5% and contains 70 ORFs. The analysis of genome variations indicated that the isolate strain showed high variations with other abovementioned strains, including substitutions, insertions, and/or deletions that occurred in most proteins, revealing that the PRV strain in southern China is quite different from previous ones.

Additionally, the main antigen of gB, gC, and gD genes which induces the neutralization antibody is important for protecting PRV infection, and the genes of gE, gI, and TK are related to virus virulence [22, 23]. Our study showed that FJ-2012 has 96.9–97.4%, 93.1–93.3%, and 97.5–98.0% sequence identity with the 7 compared strains on gB, gC, and gD, respectively (see Table S1), while the sequence identities on gE, gI, and TK are 95.8–96%, 94.2–94.5%, and 99.4–99.7%, respectively. Moreover, the US1 protein, which functions as an accessory regulatory protein and interacts with the IE protein to enhance gene transactivation [20], and the UL36 protein, which is thought to function both in early infection and in later stages of viral maturation [24], showed the highest variability between genotype I and II PRV strains.

The envelope of PRV virion, glycoprotein N protein, is encoded by UL49.5 gene, a small O-glycosylated protein that forms a disulfide-linked complex with gM and functions in viral immune evasion [25, 26]. In veterinary varicelloviruses (PRV, EHV-1, and BHV-1), the UL49.5 gene product is an inhibitor of TAP, the transporter associated with processing antigens into peptides for presentation by major histocompatibility complex (MHC) class I molecules at the cell surface [26]. In this study, gN only shares 87.9% identity with the vaccine strain Bartha and shares high homology with other Chinese PRV strains (Table 2, Figure 3). The great variations between vaccine strains and novel Chinese strains might be a potential source of the novel isolate PRV strains to evade the immune response.

Finally, the apparent genetic relationships among PRVs and the sufficient genomic variants in the field isolate strain FJ-2012 are identified to distinguish it from 2 major genotypes based on phylogenetic analysis, which might be a factor contributing to the vaccination failure in a vaccinated farm.

## 5. Conclusions

Since late 2011, pseudorabies outbreaks have been reported and spread in many farms in China. However, all novel isolated PRV genome reports were isolated in northern China, and there is no systematic research in southern China. In this study, we observed that the PRV FJ-2012 isolated from Fujian Province, southern China, is highly pathogenic and has extensive variation in genomic sequence with the reference PRV. This study contributes to the study of epidemiology and genetic evolution on PRV and lays a scientific foundation for the development of new PR vaccine.

## Supplementary Material

Table S1: Comparison of PRV FJ-2012 with other strains on specific genomic regions. a Length of ORF in codons. b %Id, percent amino acid. r indicates ORF encoded on reverse strand.

## Figures and Tables

**Table 1:** The outcome of the pigs inoculated intranasally (i.n.) with FJ-2012 strain or DMEM.

Groups	Dose (TCID50) 1 mL	Survival rate (day post infection)									Mortality (%)
		6	7	8	9	10	11	12	13	14	
PRV FJ-2012	10^6^	4/4	4/4	3/4	3/4	2/4	2/4	1/4	1/4	0/4	100
Cohabitation	DMEM	2/2	2/2	2/2	2/2	2/2	2/2	1/2	1/2	1/2	50
Control	DMEM	4/4	4/4	4/4	4/4	4/4	4/4	4/4	4/4	4/4	0

**Table 2:** Comparison of PRV FJ-2012 with other strains.

Genome	Kaplan	Becker	Bartha	JS-2012	HeN1	TJ	ZJ01
Identity (%)	91.89	91.44	89.82	96.84	96.82	98.32	94.17
Total changes	11,893	12,621	14,956	4649	4628	2445	8557
SNP	3759	3724	3789	716	560	206	1025
