# Supplementary material for: Pathogenicity and Whole Genome Sequence Analysis of a Pseudorabies Virus Strain FJ-2012 Isolated from Fujian, Southern China
Source: Can J Infect Dis Med Microbiol. 2017 Dec 31;2017:9073172. doi: 10.1155/2017/9073172 (PMC5804112; doi:10.1155/2017/9073172)
Supplement: Supplementary file 1 — Table S1: Comparison of PRV FJ-2012 with other strains on specific genomic regions. a Length of ORF in codons. b %Id, percent amino acid. r indicates ORF encoded on reverse strand. [file 9073172.f1.docx]

**Table S1. Comparison PRV FJ-2012 with other strains on specific genomic regions**

| Gene | Position  （length）^a^ | Kaplan | | Becker | | Bartha | | HeN1 | | TJ | | ZJ01 | | JS2012 | |
| --- | --- | --- | --- | --- | --- | --- | --- | --- | --- | --- | --- | --- | --- | --- | --- |
|  |  | ORF | %Id^b^ | ORF | %Id^b^ | ORF | %Id^b^ | ORF | %Id^b^ | ORF | %Id^b^ | ORF | %Id^b^ | ORF | %Id^b^ |
| ORF-1 | 1581-2204(207) | ORF-1 | 94.9 | UL56 | 95.2 | UL56 | 94.9 | UL56 | 99.8 | UL56 | 99.8 | UL56 | 99.8 | UL56 | 99.7 |
| UL54 | 2891-3976(361)r | UL54 | 96.7 | ICP22 | 97.0 | ICP22 | 96.1 | UL54 | 99.4 | ICP22 | 100.0 | ICP22 | 100.0 | ICP22 | 98.9 |
| UL53 | 4055-4993(312)r | UL53 | 96.5 | gk | 96.2 | gk | 96.5 | UL53 | 100.0 | gk | 100.0 | gk | 100.0 | gk | 99.4 |
| UL52 | 4948-7854(968)r | UL52 | 96.4 | UL52 | 96.4 | UL52 | 96.2 | UL52 | 99.7 | UL52 | 99.9 | UL52 | 98.7 | UL52 | 100.0 |
| UL51 | 7841-8572(243) | UL51 | 93.6 | UL51 | 93.2 | UL51 | 93.6 | UL51 | 100.0 | UL51 | 100.0 | UL51 | 100.0 | UL51 | 100.0 |
| UL50 | 8772-9581(269)r | UL50 | 97.9 | dUTPase | 98.0 | dUTPase | 97.9 | UL50 | 100.0 | dUTPase | 100.0 | dUTPase | 99.9 | dUTPase | 99.8 |
| UL49.5 | 9502-9801(99) | UL49.5 | 88.9 | gN | 88.9 | gN | 87.9 | UL49.5 | 100.0 | gN | 100.0 | gN | 100.0 | gN | 99.0 |
| UL49 | 9839-10579(246) | UL49 | 96.3 | VP22 | 95.5 | VP22 | 96.3 | UL49 | 100.0 | VP22 | 100.0 | VP22 | 100.0 | VP22 | 99.6 |
| UL48 | 10643-11884(413) | UL48 | 98.1 | VP16 | 98.3 | VP16 | 97.6 | UL48 | 100.0 | VP16 | 100.0 | UL48 | 100.0 | VP16 | 99.5 |
| UL47 | 11993-14209(738) | UL47 | 96.3 | VP13/14 | 95.6 | VP13/14 | 95.2 | UL47 | 99.7 | VP13/14 | 100.0 | VP13/14 | 99.6 | VP13/14 | 100.0 |
| UL46 | 14228-16315(695) | UL46 | 93.4 | VP11/12 | 93.3 | VP11/12 | 93.1 | UL46 | 99.9 | VP11/12 | 100.0 | VP11/12 | 99.6 | VP11/12 | 100.0 |
| UL27 | 16710-19454(914)r | UL27 | 97.4 | gB | 97.5 | gB | 96.9 | UL27 | 100.0 | gB | 100.0 | gB | 99.9 | gB | 100.0 |
| UL28 | 19325-21496(723)r | UL28 | 98.7 | ICP18.5 | 98.1 | ICP18.5 | 98.9 | UL28 | 99.9 | ICP18.5 | 100.0 | UL28 | 100.0 | ICP18.5 | 99.2 |
| UL29 | 21641-25183(1180)r | UL29 | 99.2 | ICP8 | 99.1 | ICP8 | 99.2 | UL29 | 100.0 | ICP8 | 100.0 | UL29 | 99.9 | ICP8 | 100.0 |
| UL30 | 25482-28628(1048) | UL30 | 99.3 | UL30 | 99.3 | UL30 | 99.3 | UL30 | 99.9 | UL30 | 100.0 | UL30 | 99.9 | UL30 | 100.0 |
| UL31 | 28549-29364(271)r | UL31 | 98.2 | UL31 | 98.2 | UL31 | 98.2 | UL31 | 100.0 | UL31 | 100.0 | UL31 | 100.0 | UL31 | 100.0 |
| UL32 | 29357-30772(471)r | UL32 | 98.7 | UL32 | 98.7 | UL32 | 98.3 | UL32 | 100.0 | UL32 | 100.0 | UL32 | 100.0 | UL32 | 100.0 |
| UL33 | 30771-31127(118) | UL33 | 95.7 | UL33 | 95.7 | UL33 | 95.7 | UL33 | 99.2 | UL33 | 99.2 | UL33 | 99.2 | UL33 | 98.3 |
| UL34 | 31299-32084(261) | UL34 | 94.7 | UL34 | 95.0 | UL34 | 94.7 | UL34 | 100.0 | UL34 | 100.0 | UL34 | 100.0 | UL34 | 99.2 |
| UL35 | 32139-32450(103) | UL35 | 98.1 | UL35 | 98.1 | UL35 | 97.1 | UL35 | 100.0 | UL35 | 100.0 | VP26 | 100.0 | VP26 | 100.0 |
| UL36 | 32953-42534(3193)r | UL36 | 91.3 | VP1/2 | 91.6 | VP1/2 | 91.6 | UL36 | 99.0 | VP1/2 | 100.0 | UL36 | 96.2 | VP1/2 | 99.9 |
| UL37 | 42572-45331(919)r | UL37 | 96.6 | UL37 | 96.6 | UL37 | 96.6 | UL37 | 99.9 | UL37 | 100.0 | UL37 | 100.0 | UL37 | 99.7 |
| UL38 | 45388-46494(368) | UL38 | 99.2 | VP19c | 99.2 | VP19c | 99.2 | UL38 | 100.0 | VP19c | 100.0 | VP19c | 100.0 | VP19c | 99.7 |
| UL39 | 46831-49197(788) | UL39 | 98.5 | RR1 | 98.5 | RR1 | 98.2 | UL39 | 100.0 | UL39 | 100.0 | RR1 | 99.7 | RR1 | 100.0 |
| UL40 | 49207-50118(303) | UL40 | 100.0 | RR2 | 98.0 | RR2 | 99.7 | UL40 | 100.0 | RR2 | 100.0 | RR2 | 100.0 | RR2 | 99.7 |
| UL41 | 50684-51781(365)r | UL41 | 98.6 | VHS | 98.6 | VHS | 98.4 | UL41 | 100.0 | VHS | 100.0 | UL41 | 100.0 | VP19c | 100.0 |
| UL42 | 51910-53067(385) | UL42 | 98.4 | UL42 | 98.4 | UL42 | 98.2 | UL42 | 100.0 | UL42 | 100.0 | UL42 | 99.7 | UL42 | 100.0 |
| UL43 | 53214-54248(344) | UL43 | 98.8 | UL43 | 99.1 | UL43 | 98.6 | UL43 | 100.0 | UL43 | 100.0 | UL43 | 100.0 | UL43 | 100.0 |
| UL44 | 54315-55778(487) | gC | 93.3 | gC | 93.3 | gC | 93.1 | UL44 | 100.0 | gC | 100.0 | gC | 100.0 | gC | 100.0 |
| UL26 | 56074-57672(532)r | UL26.5 | 99.0 | UL26.5 | 98.9 | UL26.5 | 99.0 | UL26.5 | 100.0 | UL26.5 | 100.0 | UL26.5 | 100.0 | UL26.5 | 97.9 |
| UL26.5 | 56074-56934(286)r | UL26 | 98.6 | VP24 | 98.2 | VP24 | 98.6 | VP24 | 100.0 | UL26 | 100.0 | VP24 | 100.0 | UL26 | 100.0 |
| UL25 | 57724-59349(536)r | UL25 | 98.5 | UL25 | 98.9 | UL25 | 98.9 | UL25 | 99.6 | UL25 | 100.0 | UL25 | 100.0 | UL25 | 99.8 |
| UL24 | 59433-59948(171)r | UL24 | 98.8 | UL24 | 98.8 | UL24 | 98.8 | UL24 | 98.8 | UL24 | 100.0 | UL24 | 100.0 | UL24 | 100.0 |
| UL23 | 59941-60903(320) | UL23 | 99.7 | TK | 99.4 | TK | 99.4 | UL23 | 100.0 | TK | 100.0 | TK | 100.0 | TK | 100.0 |
| UL22 | 61039-63096(685) | UL22 | 99.4 | gH | 98.8 | gH | 99.6 | UL22 | 99.1 | UL22 | 99.7 | gH | 99.9 | UL22 | 99.6 |
| UL21 | 65017-66618(533)r | UL21 | 96.9 | UL21 | 96.8 | UL21 | 96.4 | UL21 | 99.8 | UL21 | 99.8 | UL21 | 96.8 | UL21 | 99.8 |
| UL20 | 66725-67222(165) | UL20 | 95.7 | UL20 | 95.7 | UL20 | 95.7 | UL20 | 100.0 | UL20 | 100.0 | UL20 | 100.0 | UL20 | 100.0 |
| UL19 | 67311-71303(1330) | UL19 | 99.4 | VP5 | 99.3 | VP5 | 99.3 | UL19 | 99.8 | VP5 | 99.9 | VP5 | 99.8 | VP5 | 99.8 |
| UL18 | 71477-72424(315) | UL18 | 99.3 | VP23 | 99.3 | VP23 | 99.3 | UL18 | 100.0 | VP23 | 100.0 | UL18 | 100.0 | VP23 | 99.0 |
| UL15(Ex2) | 72549-73754(401)r | UL15 | 95.5 | UL15 | 95.3 | UL15 | 95.1 | UL15 | 99.9 | UL15 | 100.0 | UL15 | 99.6 | UL15 | 99.7 |
| UL15(Ex1) | 76535-77686(383)r |  |  |  |  |  |  |  |  |  |  |  |  |  |  |
| UL17 | 73741-75540(599) | UL17 | 96.7 | UL17 | 96.3 | UL17 | 96.0 | UL17 | 100.0 | UL17 | 100.0 | UL17 | 98.7 | UL17 | 97.8 |
| UL16 | 75567-76538(323) | UL16 | 93.5 | UL16 | 92.9 | UL16 | 93.5 | UL16 | 98.1 | UL16 | 98.1 | UL16 | 98.1 | UL16 | 98.1 |
| UL14 | 77685-78164(159) | UL14 | 96.2 | UL14 | 96.2 | UL14 | 95.6 | UL14 | 99.4 | UL14 | 99.4 | UL14 | 99.4 | UL14 | 99.4 |
| UL13 | 78134-79309(391) | UL13 | 95.4 | VP18.8 | 95.7 | VP18.8 | 95.7 | UL13 | 100.0 | VP18.8 | 100.0 | UL13 | 98.7 | VP18.8 | 100.0 |
| UL12 | 79275-80726(483) | UL12 | 96.1 | AN | 95.9 | AN | 96.1 | UL12 | 99.8 | AN | 99.8 | UL12 | 99.6 | AN | 100.0 |
| UL11 | 80684-80875(63) | UL11 | 98.4 | UL11 | 96.9 | UL11 | 96.9 | UL11 | 100.0 | UL11 | 100.0 | UL11 | 100.0 | UL11 | 100.0 |
